# Yttrium oxide nanoparticles ameliorates calcium hydroxide and calcium titanate nanoparticles induced genomic DNA and mitochondrial damage, ROS generation and inflammation

**DOI:** 10.1038/s41598-024-62877-4

**Published:** 2024-06-06

**Authors:** Hanan R. H. Mohamed, Ahmed H. Farouk, Salma H. Elbasiouni, Kirolls A. Nasif, Gehan Safwat

**Affiliations:** 1 Zoology Department, Faculty of Science, Cairo University, Giza, Egypt; 2 Faculty of Biotechnology, October University for Modern Sciences and Arts, 6 Ocober, Egypt

**Keywords:** Genotoxicity, Nanoparticles, Calcium hydroxide, Calcium titanate, Yttrium oxide, ROS generation, Mitochondrial membrane potential, Natural hazards, Health occupations, Risk factors

## Abstract

Calcium hydroxide (Ca(OH)_2_NPs), calcium titanate (CaTiO_3_NPs) and yttrium oxide (Y_2_O_3_NPs) nanoparticles are prevalent in many industries, including food and medicine, but their small size raises concerns about potential cellular damage and genotoxic effects. However, there are very limited studies available on their genotoxic effects. Hence, this was done to investigate the effects of multiple administration of Ca(OH)_2_NPs, CaTiO_3_NPs or/and Y_2_O_3_NPs on genomic DNA stability, mitochondrial membrane potential integrity and inflammation induction in mouse brain tissues. Mice were orally administered Ca(OH)_2_NPs, CaTiO_3_NPs or/and Y_2_O_3_NPs at a dose level of 50 mg/kg b.w three times a week for 2 weeks. Genomic DNA integrity was studied using Comet assay and the level of reactive oxygen species (ROS) within brain cells was analyzed using 2,7 dichlorofluorescein diacetate dye. The expression level of Presenilin-1, tumor necrosis factor-alpha (TNF-α) and Interleukin-6 (IL-6) genes and the integrity of the mitochondrial membrane potential were also detected. Oral administration of Ca(OH)_2_NPs caused the highest damage to genomic DNA and mitochondrial membrane potential, less genomic DNA and mitochondrial damage was induced by CaTiO_3_NPs administration while administration of Y_2_O_3_NPs did not cause any remarkable change in the integrity of genomic DNA and mitochondrial membrane potential. Highest ROS generation and upregulation of presenilin-1, TNF-α and IL-6 genes were also observed within the brain cells of mice administrated Ca(OH)_2_NPs but Y_2_O_3_NPs administration almost caused no changes in ROS generation and genes expression compared to the negative control. Administration of CaTiO_3_NPs alone slightly increased ROS generation and the expression level of TNF-α and IL-6 genes. Moreover, no remarkable changes in the integrity of genomic DNA and mitochondrial DNA potential, ROS level and the expression level of presenilin-1, TNF-α and IL-6 genes were noticed after simultaneous coadministration of Y_2_O_3_NPs with Ca(OH)_2_NPs and CaTiO_3_NPs. Coadministration of Y_2_O_3_NPs with Ca(OH)_2_NPs and CaTiO_3_NPs mitigated Ca(OH)_2_NPs and CaTiO_3_NPs induced ROS generation, genomic DNA damage and inflammation along with restoring the integrity of mitochondrial membrane potential through Y_2_O_3_NPs scavenging free radicals ability. Therefore, further studies are recommended to study the possibility of using Y_2_O_3_NPs to alleviate Ca(OH)_2_NPs and CaTiO_3_NPs induced genotoxic effects.

## Introduction

Nanotechnology is a cutting-edge field of science that deals with the engineering and study of technology at a nanoscale. The term “nano” refers to a unit of measurement that is one thousandth of a micro and demonstrates the minuscule size of these particles. Nanoparticles are incredibly tiny, measuring more than 8000 times smaller than a human hair^1^. This technology is incredibly versatile and has many applications, as it continues to grow as a field. It is crucial to study its effects on biological organisms to prevent any potential harm from the nanoparticles used in a wide range of industries, such as healthcare, food preservation, and cosmetics. Nanotechnology has a profound effect on every aspect of our daily lives, from enhancing security to advancing medicine^2^.

Sadly, advances in the production and applications of nanoparticles have led to a rise in human exposure to several manufactured nanoparticles such as Calcium hydroxide (Ca(OH)_2_NPs), Calcium titanate (CaTiO_3_NPs), and Yttrium oxide (Y_2_O_3_NPs) nanoparticles^3^. The very unique traits of Ca(OH)_2_NPs highly raise their uses in various nanotechnological and biotechnology applications: One of them is their ability to protect limestone's statues in their aqueous form and in this cane they are known as nanolime^4^. The initial studies on Ca(OH)_2_NPs as nanolime by scientists took place around 2000 at the University of Florence CSGI in Italy. The results of their research on its synthesis and use for preserving wall paintings were published in 2001^4^. Moreover, CaTiO_3_NPs are also heavily used in fresh water and wastewater treatment to raise the water PH and remove particles from water in addition to their uses as a potent antimicrobial in intra-cranial medicine^3,4^.

Calcium titanite is a compound made up of calcium (Ca) and titanium (Ti). It can be produced through several techniques, including sol–gel, coprecipitation, hydrothermal, mechanochemical, solid-state, and co-precipitation. The sol–gel method is the most commonly used method for producing calcium titanite^5^. CaTiO_3_NPs are widely employed in a variety of applications, including electronic ceramics, energy storage, and biological imaging^6^. Despite the obvious advantages of CaTiO_3_NPs, they offer certain health and environmental risks. They have the potential to generate oxidative stress and DNA damage, which could result in toxicity in normal Human skin fibroblast Cells^7^. If released into the water system, they can also potentially be hazardous to aquatic life.

Nanoparticles of Yttrium oxide have a wide range of applications due to their unique properties. For example Y_2_O_3_NPs are used as a polarizer, laser host material, phosphor, bio-imaging, and biosensor, along with their uses in the treatment of cancer and Fulminant hepatic failure. Despite this, Y_2_O_3_NPs alone have not gained much attention as a nanomedicine^8,9^. Therefore, the present study was undertaken to estimate the effect of multiple oral administration with Ca(OH)_2_NPs, CaTiO_3_NPs or/and Y_2_O_3_NPs on the integrity of genomic and mitochondrial DNA in mice brain tissues. Alkaline Comet assay was done to assess genomic DNA integrity, while integrity of mitochondrial membrane potential was studied using 3-Rhodamine dye. The level of reactive oxygen species (ROS) generation within neural cells was studied using 2,7-dichlorofluorescein dye and expression level of presenilin-1 gene and inflammatory tumor necrosis factor-alpha (TNF-α) and interleukin-6 (IL-6) genes was measured using quantitative Real time PCR.

## Materials and methods

### Chemicals

The Ca(OH)_2_NPs were procured from Nanotech Company (Giza, Egypt), while CaTiO_3_NPs were sourced from Sigma Aldrich Company (St. Louis, MO, USA) with the product code 633801. These particles are in the form of Nano powder with a particle size less than 100 nm and contain 99% trace metals. Y_2_O_3_NPs were also obtained from Sigma-Aldrich Company (St. Louis, MO, USA) with product number 544892 and in the form of nano-powders with a particle size of less than 50 nm and contain at least 99.9% trace metals.

### Characterization of nanoparticles

The three tested nanoparticles: Ca(OH)_2_NPs, CaTiO_3_NPs and Y_2_O_3_NPs have been characterized well in our previous studies using transmission electron microscope (TEM), X-ray diffraction (XRD) and dynamic laser scattering (DLS)^7,10,11^.

### Animals

This study involved using 45 male Swiss Webster mice, aged 10–12 weeks and weighing 20–25 g. The mice were purchased from the National Research Center and kept in normal conditions for 1 week prior starting the administration at the animal house of Zoology Department at Faculty of Science Cairo University. They were fed standard diet pellets and water. Mice were fed completely nutritional diet called Mazuri's vegetarian rat and mouse diet manufuctured by Land O' Lakes Inc Company (Arden Hills, Minnesota, USA).

### Ethical approval

The design of this study was approved by the MSA University Research Ethics Committee. This study was reported according to ARRIVE guidelines and also Animal handling and experimentations were conducted in accordance with the Guidelines of the National Institutes of Health (NIH) regarding the care and use of animals for experimental procedures.

### Determination of the nanoparticles' tested dose

An acute toxicity test was used to determine the appropriate utilized dose of the tested nanoparticles according to OECD standards 420 as follow: Twenty male mice were divided into four groups: an untreated control group and three treated groups, each with five mice. The three treated groups were orally given 2000 mg/kg of Ca(OH)_2_NPs, CaTiO_3_NPs or Y_2_O_3_NPs separately, while mice of the negative control group were orally given deionized distilled water. All mice of the four groups were monitored for any symptoms or morphological behavior of toxicity during the first 24 h of nanoparticles administration and up to 14 days of administration. Based on the mice's survival and the OECD standards 420^12,13^, the used dose of the tested nanoparticles was 2.5% of the safety tested dose determined from the OECD test.

### Experimental design

In this study, 25 male mice were randomly separated into five groups with 5 mice each (Fig. S1). The first group served as the negative control, while the second group was taken 50 mg/kg of Ca(OH)_2_NPs through oral administration, the third group was given 50 mg/kg of CaTiO_3_NPs orally, the fourth group was given 50 mg/kg of Y_2_O_3_NPs, and the fifth group was given a combination of the tested nanoparticles orally at a dose level of 50 mg/kg each, three times a week for two consecutive weeks. After 24 h of the last administration, all mice of the five groups were put to death by cervical dislocation, dissected and their brains were extracted, frozen, and preserved at − 80 °C for further analysis.

### Detection of reactive oxygen species generation

The generation of reactive oxygen species (ROS) within brain cells was detected using 2,7 dichlorofluorescein diacetate (DCFH-DA) according to^14^. This compound can penetrate cells and react with ROS to produce the fluorescent chemical dichlorofluorescein (DCF). The procedure involved homogenizing 50 mg of brain tissue in Phosphate buffered saline (PBS), then rinsing it twice with PBS. 50 μl of the cell suspension was mixed with 50 μl of DCFH-DA (20 mM) and left to incubate in the dark for 30 min. The mixture was then placed on slides and imaged under a fluorescent microscope (OLYMPUS CKX 41) at 20× magnification.

### Estimation of DNA damage level

The level of DNA damage induction within brain cells was measured using the alkaline Comet test with a pH level higher than 13^15^. Slides were dipped in normal melting agarose (1%) and the samples were gently minced, mixed with low melting agarose (0.5%) and then placed on coated normal agarose slides. Sides were kept in the dark for 24 h at 4 °C in cold lysis buffer. After lysis, the slides were placed in staining jars containing alkaline electrophoresis buffer for 15 min, then electrophoresed for 30 min at 25 V and 300 mA in the same alkaline buffer. Slides were then neutralized, fixed with cold absolute ethanol and stained with ethidium bromide prior imaging. The slides were finally photographed under an epi-fluorescent microscope at 200× magnification and TriTek Comet ScoreTM Freeware v1.5 was used to assess the extent of DNA damage by measuring tail length, %DNA in tail, and tail moment.

### Studying the mitochondrial membrane potential

The integrity of mitochondrial membrane potential was studied in brain tissues of the five groups using the method previously described by Zhang and his colleagues^16^. Briefly: a suspension of brain cells in PBS was combined with the fluorescent Rhodamine-123 dye and incubated in the dark for an hour at 37 °C. After incubation, the cells were washed twice with PBS. Then the fluorescence light emitted by Rhodamine-123 was captured and analyzed using an epifluorescence microscope at 200× magnification.

### mRNA expression levels of presenilin-1 and inflammatory genes

For measuring the mRNA expression level of presenilin-1 gene and inflammatory tumor necrosis factor-alpha (TNF-α) and interleukin-6 (IL-6) genes, quantitative RTPCR was conducted. The Gene JET RNA Purification Kit was used to extract RNA from frozen brain tissues in a 1.5 ml micro-centrifuge tube. About 30 mg of brain tissues were mixed with 300 µl of lysis buffer and β-mercaptoethanol and vortexed for 10 s, then 600 μl of diluted proteinase K was added, vortexed and incubated for 10 min at 25 °C. The tubes were centrifuged for 10 min at 12,000×*g*, and then the supernatant was transferred to a new RNase-free micro-centrifuge tube. 450 μl of 100% ethanol was added; the lysate solution was filtered through a GeneJet RNA Purification column and washed with washing buffer. Finally, 100 μl of nuclease-free water was introduced to the column, centrifuged for 60 s, and the RNA was eluted into a new micro-centrifuge tube, which was stored at − 80 °C. The RNA was then reverse transcribed into complementary DNA (cDNA) using the Revert Aid First Strand cDNA Synthesis Kit. For amplification, 1 μl of the cDNA of each sample for each gene was mixed with 0.5 µl of each forward and reverse primers listed in Table 1^17,18^ for three genes (Presenilin, TNF-α and IL-6) along with 6 µl of SYBER green master mix and 4 μl of nuclease-free water to make a total of 12 μl. The RTPCR reaction was then run with an initial denaturation of 95 °C for 15 min followed by 35 cycles of denaturation at 95 °C for 15 s and annealing at for 30 s, and extension at 72 °C for 1 min. Then final extension was done at 72 °C for 10 min. The expression of the three studied genes was measured using β-actin gene as a housekeeping gene and the comparative ΔΔCt method was used for calculating the fold change in the gene expression.

**Table 1:** Sequences of the used primers in qRT-PCR.

Gene	Strand	Sequence
TNF-α	Forward	5′ CCC GAG TGA CAA GCC TGT AG 3′
	Reverse	5′ GAT GGC AGA GAG GAG GTT GAC 3′
IL-6	Forward	5′ CAT GTT CTC TGG GAA ATC GTGG 3′
	Reverse	5′ AAC GCA CTA GGT TTG CCG AGTA 3′
Presenilin-1	Forward	5′ AAA GGT CCA CTT CGA CTC CA 3′
	Reverse	5′ GGC ATT CCT GTG ACA AAC AA 3′
β-actin	Forward	5′ TCA CCC ACA CTG TGC CCA TCT ACGA 3′
	Reverse	5′ GGA TGC CAC AGG ATT CCA TAC CCA 3′

### Statistical analysis

The findings from this study were displayed as mean ± SD and evaluated using SPSS (version 20) at a significance level of < 0.05. One-way analysis of variance (ANOVA) was used to determine the impact of Y_2_O_3_NPs coadministration with Ca(OH)_2_NPs and CaTiO_3_NPs on induction of DNA damage and expression level of presenilin-1, TNF-α and IL-6 genes. Duncan's test was carried out to determine the similarities and differences between the control and four treated groups.

## Results

### Characterization of nanoparticles

Characterization of Ca(OH)_2_NPs, CaTiO_3_NPs and Y_2_O_3_NPs in our previous studies using XRD analysis, DLS and TEM confirmed the purity of purchased nanopowders along with stability and well distribution of suspended nanoparticles in deionized distilled water. Moreover, TEM imaging revealed the spherical morphology of Ca(OH)_2_NPs, CaTiO_3_NPs and Y_2_O_3_NPs with an average particles' size of 59.82, 88.79 and 14.00 nm, respectively^7,10,11^.

### The nanoparticles' tested dose

Observation of mice administered orally with a single dose (2000 mg/kg b.w) of Ca(OH)_2_NPs, CaTiO_3_NPs or Y_2_O_3_NPs revealed that all mice were healthy and no signs of toxicity noticed during the first 48 h of Ca(OH)_2_NPs, CaTiO_3_NPs or Y_2_O_3_NPs administration until the end of the 14-days observation period. The half lethality dose (LD50) of Ca(OH)_2_NPs, CaTiO_3_NPs and Y_2_O_3_NPs above 2000 mg/kg was considered according to the OECD-420 guidelines, and the initial tested dose of Ca(OH)_2_NPs, CaTiO_3_NPs and Y_2_O_3_NPs in this study was calculated as 2½% (50 mg/kg body weight) of the LD50 Obtained from acute toxicity test.

### Generation of intracellular ROS

**Figure 1:**
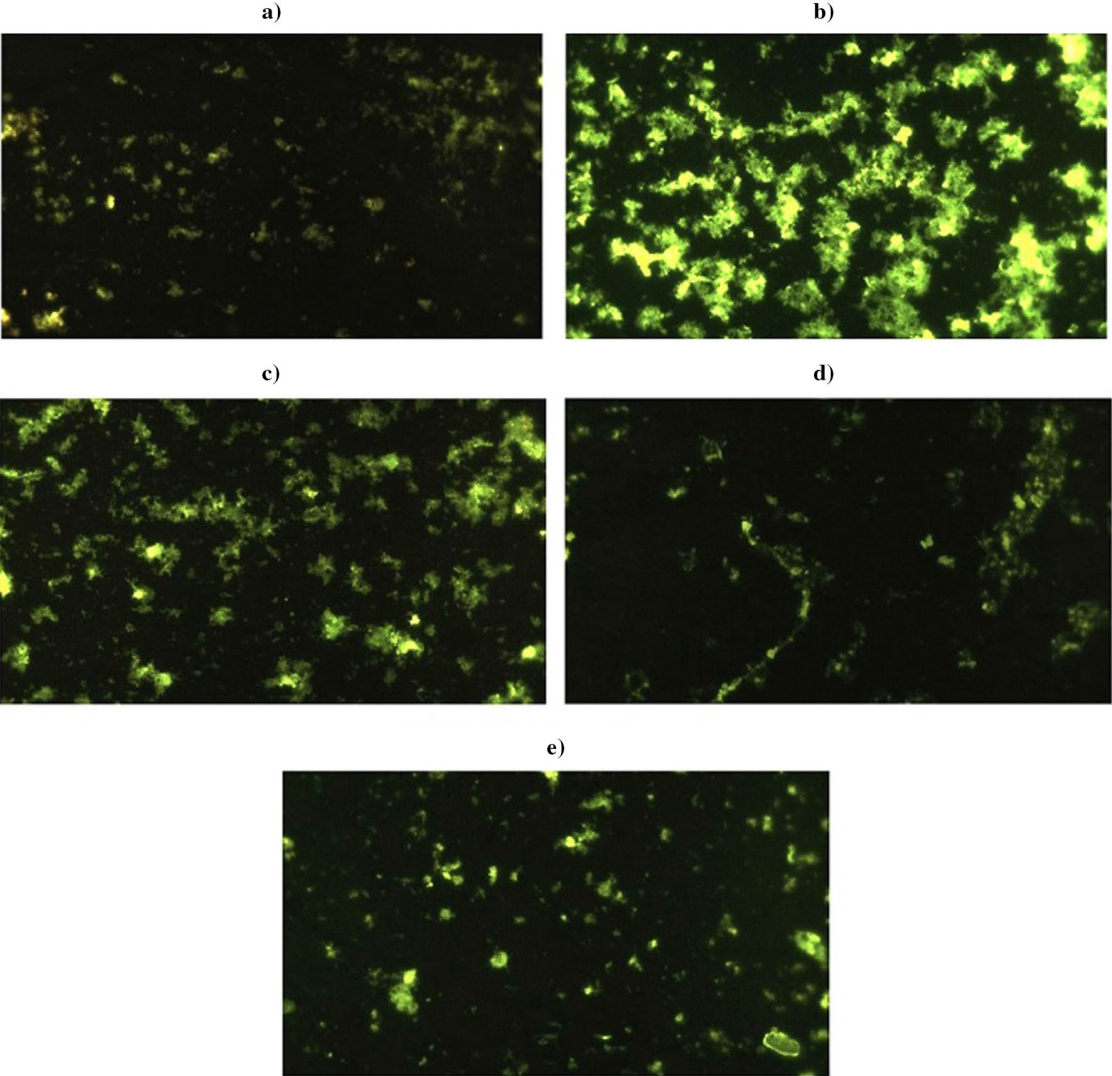
ROS level using 2,7-DCFH-DA dye within the brain cells of (**a**) Negative control group, (**b**) Ca(OH)_2_NPs administered group, (**c**) CaTiO_3_NPs administered group, (**d**) Y_3_O_2_NPs administered group and (**e**) group administered Ca(OH)_2_NPs, CaTiO_3_NPs and Y_3_O_2_NPs simultaneously.

Staining of brain cells with 2,7 DCFH-DA was highly informative and revealed that the highest generation of ROS was seen in the brain tissues of mice orally administered Ca(OH)_2_NPs alone compared to negative control and three treated groups (Groups III, IV and V) as depicted in Fig. 1. A slightly higher amount of ROS was observed in the brain tissue of mice orally ingested CaTiO_3_NPs alone compared to those noticed in the brain cells of negative control group. The last two groups administered Y_2_O_3_NPs alone or in combination with Ca(OH)_2_NPs and CaTiO_3_NPs exhibited the lowest amount of ROS generation in comparison to the other treated groups and almost identical to the ROS generated in the negative control brain cells, as displayed in Fig. 1.

### Induction of DNA damage

**Figure 2:**
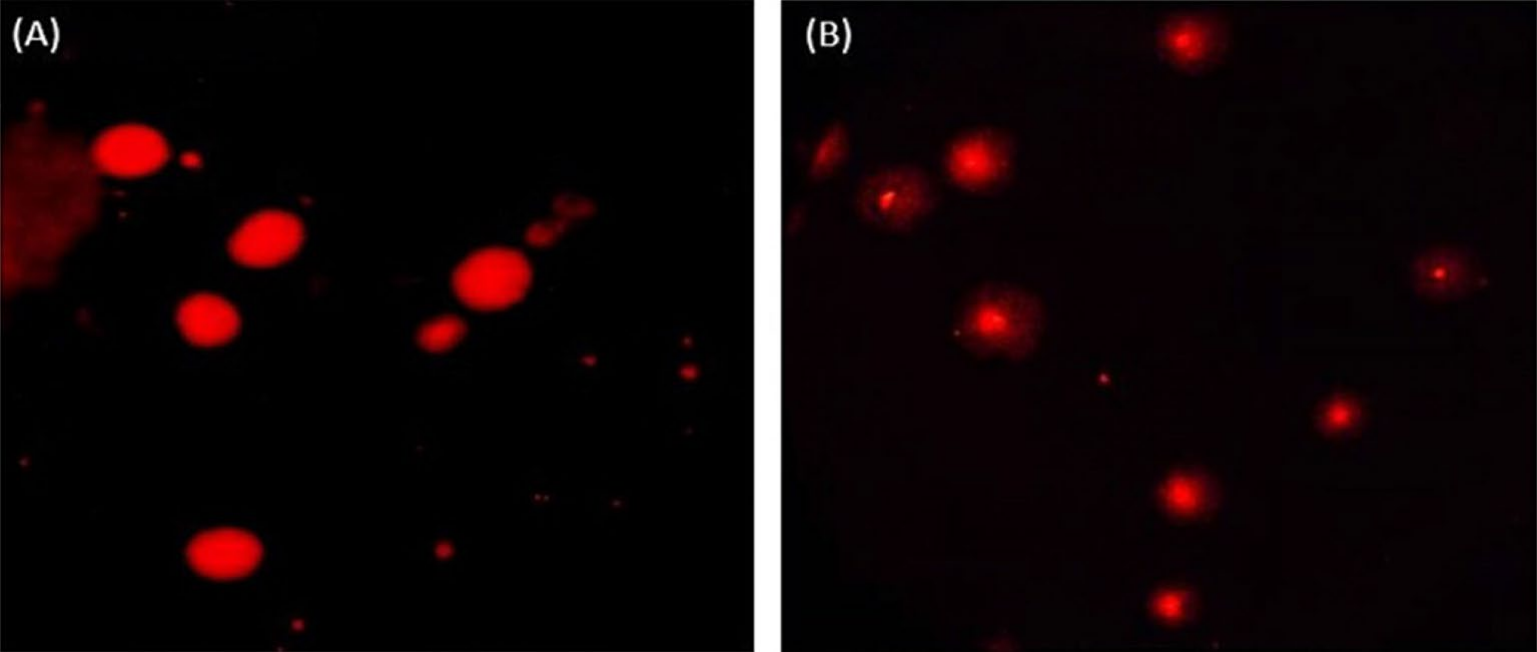
Representative photomicrograph for the observed Comet intact and damaged nuclei in the brain tissues of negative control group and Ca(OH)_2_NPs, CaTiO_3_NPs or/and Y_3_O_2_NPs administered groups. (**A**) Intact nuclei (**B**) Damaged nuclei.

Results of the Comet assay are shown in Table 2 and examples of the Comet nuclei scored with intact and damaged DNA are shown in Fig. 2. As depicted in Table 2, oral intake of Ca(OH)_2_NPs for six separate days over a 2-week period induced the highest statistical significant elevations in the DNA damage measured parameters: tail length, %DNA in tail, and tail moment compared to their values in the brain tissues of mice administered CaTiO_3_NPs or Y_2_O_3_NPs separately or together simultaneously with Ca(OH)_2_NPs (Table 2). Similarly, oral ingestion of CaTiO_3_NPs six times over a 2 weeks caused statistical significant increases in %DNA in tail and tail moment compared to the negative control values but remained significantly lower than the Ca(OH)_2_NPs administered group values (Table 2). On the other hand, oral administration of Y_2_O_3_NPs alone (Group IV) or simultaneously with Ca(OH)_2_NPs and CaTiO_3_NPs (Group V) did not cause any statistical changes in the tail length and tail moment compared to the negative control group values as displayed in Table 2.

**Table 2:** Tail length (px), %DNA in tail and tail moment in the brain tissues of the negative control group and groups orally administered Ca(OH)_2_NPs, CaTiO_3_NPs or/and Y_2_O_3_NPs.

Group	Treatment	Tail length (px)	%DNA in tail	Tail moment
I	Negative control	4.14 ± 1.12^a^	18.15 ± 4.74^a^	0.93 ± 0.47^a^
II	Ca(OH)_2_NPs	16.30 ± 4.41^b^	38.58 ± 2.26^b^	6.82 ± 1.45^b^
III	CaTiO_3_NPs	6.98 ± 0.85^a^	37.47 ± 4.07^b^	2.41 ± 0.16^c^
IV	Y_2_O_3_NPs	5.69 ± 0.13^a^	32.72 ± 7.44^b^	1.83 ± 0.68^a^
V	Ca(OH)_2_NPs + CaTiO_3_NPs + Y_2_O_3_NPs	5.54 ± 0.92^a^	28.23 ± 4.98^b^	1.52 ± 0.59^a^
One Way Analysis of Variance (ANOVA)		F = 16.13 p < 0.001	F = 7.31 p < 0.01	F = 15.50 p < 0.001

Results are expressed as mean ± SD.

Results were analyzed using one-way analysis of variance followed by Duncan’s test to test the similarity between the control and the four treated groups.

Means with different superscript letters indicates statistical significant difference between the compared groups in the same column.

### Integrity of mitochondrial membrane potential

**Figure 3:**
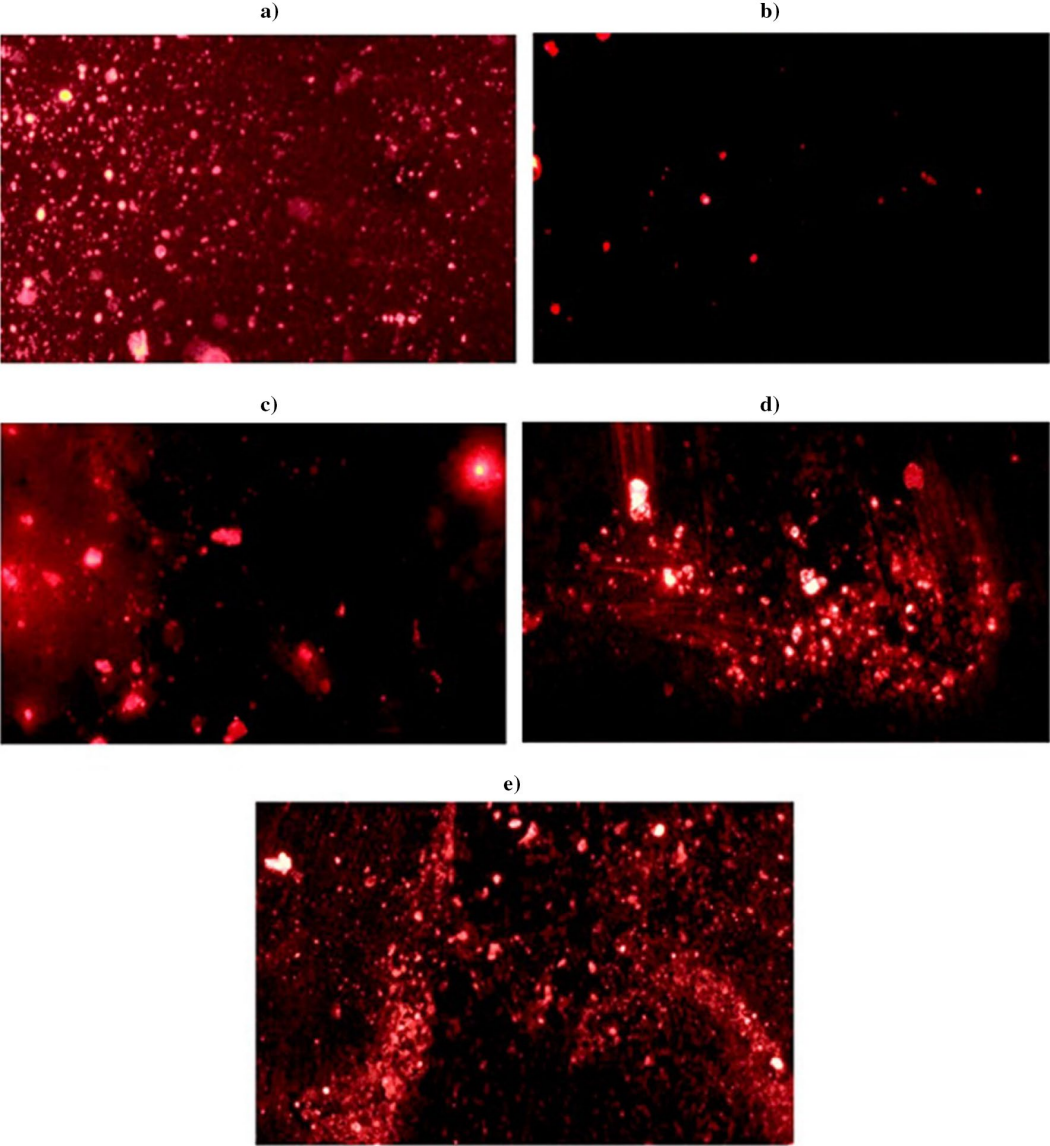
Integrity of mitochondrial membrane potential using Rhodamine dye within the brain cells of (**a**) Negative control group, (**b**) Ca(OH)_2_NPs administered group, (**c**) CaTiO_3_NPs administered group, (**d**) Y_3_O_2_NPs administered group and (**e**) group administered Ca(OH)_2_NPs, CaTiO_3_NPs and Y_3_O_2_NPs simultaneously.

As illustrated in Fig. 3, oral administration of Ca(OH)_2_NPs alone caused a highest damage to the mitochondrial membrane potential as manifested by the remarkable decrease in the fluorescence intensity emitted by Rhodamine-123 stained brain cells compared to the negative control (Group I) and other three treated groups (Groups III, IV and V). Similarly, the oral administration of CaTiO_3_NPs led to a high decrease in the intensity of emitted fluorescent light compared to that emitted from the negative control brain cells, but still higher than that emitted from brain cells of mice administered Ca(OH)_2_NPs alone.

On the other hand, minimal damage to the mitochondrial membrane potential was noticed in the brain cells of mice orally administered Y_2_O_3_NPs alone (Group IV) or in combination with Ca(OH)_2_NPs and CaTiO_3_NPs (Group V) as slight decreases in the intensity of emitted light were observed in mice given Y_2_O_3_NPs alone (Group IV) and almost no changes were seen in mice orally given Y_2_O_3_NPs with Ca(OH)_2_NPs and CaTiO_3_NPs (Group V).

### mRNA expression level

**Figure 4:**
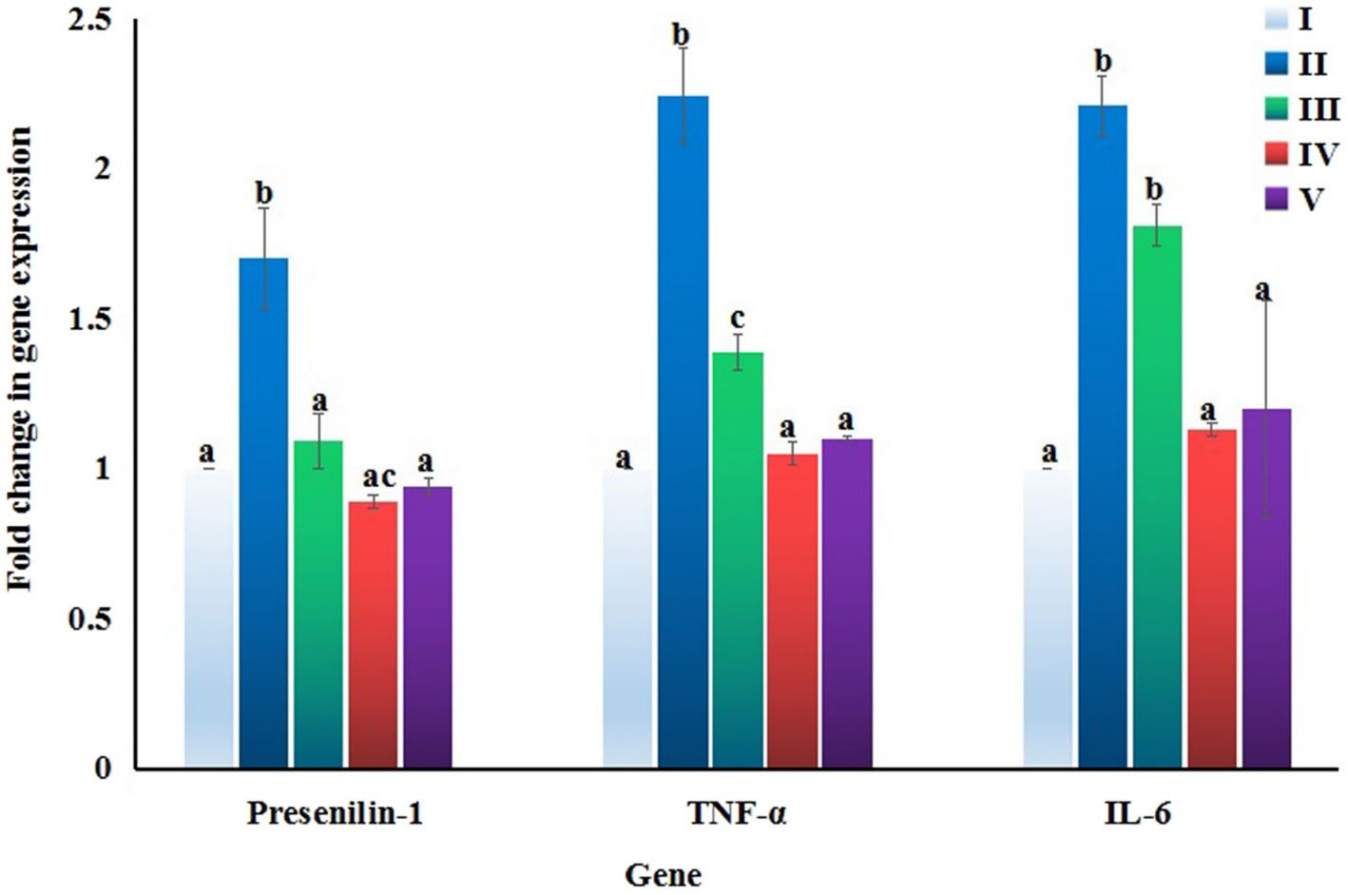
Expression level of Presenilin-1, TNF-α and IL-6 genes in in the brain tissues of the negative control group and groups orally administered Ca(OH)_2_NPs, CaTiO_3_NPs or/and Y_2_O_3_NPs. Results are expressed as mean ± SD and were analyzed using one-way analysis of variance followed by Duncan’s test to test the similarity between the control and three treated groups. Means with different superscript letters indicates statistical significant difference at p < 0.05 between the compared groups for the same gene.

The quantitative RTPCR results are summarized in Fig. 4 and showed that the expression level of the three studied genes: presenilin-1, TNF-α, and IL-6 genes was statistically significantly elevated in the brain tissues of mice orally given Ca(OH)_2_NPs compared to their expression level in the negative control and other three groups CaTiO_3_NPs or Y_2_O_3_NPs separately or together with Ca(OH)_2_NPs. On the other hand, oral administration of administration of CaTiO_3_NPs significantly upregulated the expression level of TNF-α and IL-6 genes compared to the negative control expression level but significantly less than their expression level in Ca(OH)_2_NPs administered group as seen in Fig. 4. Meanwhile no remarkable changes were observed in the expression level of presenilin-1 gene after CaTiO_3_NPs administration compared to the negative control expression level (Fig. 4). On the contrary, the expression level of presenilin-1, TNF-α, and IL-genes did not change significantly and remained at the expression level of negative control after administration of Y_2_O_3_NPs alone (Group IV) or in simultaneously with Ca(OH)_2_NPs and CaTiO_3_NPs (Group V) as depicted in Fig. 4.

## Discussion

The study of the cytotoxic and genotoxic effects of nanomaterials and nanoparticles is crucial in the field of biotechnology as nanotechnology advances. Extensive uses of Ca(OH)_2_NPs and CaTiO_3_NPs in various industrial, medical, food and consumer products increase the incidence of human exposure to these nanoparticles. However, limited data are available on the effect of Ca(OH)_2_NPs and CaTiO_3_NPs on the integrity of genomic and mitochondrial DNA in vivo along with the recently discovered free radicals scavenging activity of Y_2_O_3_NPs. Therefore, the current study was undertaken to estimate the impact of Y_2_O_3_NPs coadministration with Ca(OH)_2_NPs and CaTiO_3_NPs on the integrity of genomic DNA and mitochondrial membrane potential in the mice brain tissues.

In this study tracking ROS generation demonstrated the highest generation of ROS within the brain cells of mice orally given Ca(OH)_2_NPs (Group II) compared to those generated by CaTiO_3_NPs (Group III) or Y_2_O_3_NPs (Group IV) separately or in combination with Ca(OH)_2_NPs (Group V) as shown in Fig. 1. These results are in consistent with the recent detection of excessive ROS generation after single oral administration of Ca(OH)_2_NPs in various mice tissues: brain, bone marrow, liver, heart, spleen and lung^10,19^. Moreover, our finding of excessive ROS generation after administration of CaTiO_3_NPs manifested the in vivo induction of oxidative stress by CaTiO_3_NPs and supported the recent discovery of ROS generation and oxidative stress induction by CaTiO_3_NPs in breast cancer MCF-7 cell line^20^.

Extra-ROS generation can cause single- and double-strand DNA breaks, which is a lethal form of DNA damage^21,22^. According to Mills study^23^, double stranded-DNA breaks play a key role in the initiation of proto-oncogenes and the pathogenesis of cancer, suggesting that increased intracellular ROS production can cause cancer since intensive ROS generation disrupts the balance between oxidants and antioxidants and attacks proteins, lipids, carbohydrates and DNA inducing lipid peroxidation, protein damage and destruction, oxidative DNA damage and alterations in DNA bases. Indeed, the alkaline Comet assay is a very sensitive technique in detecting both single- and double-stranded DNA breaks^15^. Consequently, the highest incidence of DNA damage induction demonstrated by Comet assay in the brain tissues of mice given Ca(OH)_2_NPs alone compared to the other three treated groups (Table 2 and Fig. 2) can be attributed to the aforementioned highest ROS generation by Ca(OH)_2_NPs attacking DNA and cause breakages of both single and double DNA strands. Similarly, a recent study by Mohamed^10^, manifested the induction of DNA breakages in the brain tissues of mice given orally Ca(OH)_2_NPs through increased generation of intracellular ROS.

Similarly, the detected remarkable DNA damage induction by CaTiO_3_NPs administration through significant increases in the percentage of damaged DNA in the tail and tail moment could be attributed to the increased ROS generation noticed within brain cells of mice orally given CaTiO_3_NPs alone compared to those generated in the negative control brain cells. High ROS consequently attacks genomic DNA and increases the amount of damaged DNA. However, non remarkable changes observed in the tail length value after CaTiO_3_NPs administration compared to the negative control value revealed the large size of fragmented DNA causing slow migration in the mini gel^15,23^.

On the other hand, oral administration of Y_2_O_3_NPs alone caused non remarkable changes in the integrity of genomic DNA as demonstrated by the observable non-significant changes in the tail length and tail moment compared to negative control levels. Similarly, simultaneous coadministration of Y_2_O_3_NPs with Ca(OH)_2_NPs and CaTiO_3_NPs caused a marked decrease in the incidence of DNA damage induction noticed after administration of Ca(OH)_2_NPs or CaTiO_3_NPs separately and became non-statistically different from the negative control levels (Table 2). This remarkable reduction in the DNA damage induction probably due to the recently discovered antioxidant properties of Y_2_O_3_NPs; Y_2_O_3_NPs act as a direct antioxidant, controlling and neutralizing the generated harmful ROS^8,9,24^. Our findings of minimal ROS generation within brain cells of mice that were given Y_2_O_3_NPs alone or in combination with Ca(OH)_2_NPs and CaTiO_3_NPs further confirmed the antioxidant and free radicals scavenging capabilities of Y_2_O_3_NPs. Consistent with our findings, Y_2_O_3_NPs showed a potent dose dependent antioxidant and neuroprotective effect against oxidative stress and apoptosis induction^25–28^.

For more understanding of the impact of Y_2_O_3_NPs coadministration with Ca(OH)_2_NPs and CaTiO_3_NPs on the integrity of genomic DNA, the mRNA expression of Presenilin-1, TNF-α, and IL-6 genes were measured. Presenilin-1 is responsible for cleaving amyloid protein that causes Alzheimer's disease and is overexpressed in Alzheimer's patients^29–31^. Results of RTPCR showed that Y_2_O_3_NPs coadministration with Ca(OH)_2_NPs and CaTiO_3_NPs highly declined presenilin-1 overexpression (Fig. 4) noticed after oral administration of Ca(OH)_2_NPs alone thus protecting brain cells from Alzheimer's risk caused by administration of Ca(OH)_2_NPs alone previously reported by the study of Li and his colleagues^30^. Moreover, a marked decreases in the expression of presenilin-1 genes after oral administration of Y_2_O_3_NPs indicating the potentiality of Y_2_O_3_NPs in protecting the brain cells from Alzheimer's disease.

Alzheimer's and other neurological diseases have been linked to elevated inflammatory cytokines expression and secretion. For example, the expression level of IL-6 and TNF-α genes is elevated in Alzheimer's disease^30–32^. Overexpression of inflammatory mediators including IL-6 and TNF-α genes also increases the expression level of Presenilin-1 and β-amyloid precursor protein genes causing aggregation and accumulation of β-amyloid peptides in the brain tissues and increasing the risk of Alzheimer's disease^31,33^. A marked overexpression of TNF-α and IL-6 genes observed in the brain tissue of mice orally exposed to Ca(OH)_2_NPs or CaTiO_3_NPs (Fig. 4), suggesting inflammation induction and immune stimulation^19,28^. However, no significant difference was found in the expression level of TNF-α and IL-6 genes after administration of Y_2_O_3_NPs alone revealing the antioxidant and protective properties of Y_2_O_3_NPs on brain cells. Therefore, our findings regarding the remarkable high decreases observed in the expression level IL-6 and TNF-α overexpressed genes by administration of Ca(OH)_2_NPs or CaTiO_3_NPs alone may explain the restored normal gene expression of presenilin-1 after Y_2_O_3_NPs coadministration with Ca(OH)_2_NPs and CaTiO_3_NPs (Fig. 4).

Variations in mitochondrial membrane potential can assess mitochondrial function and cell health using fluorescent dyes^34^. Mitochondria are critical to cell survival and any damage leads to cell death and disease. Therefore, the integrity of mitochondrial membrane potential has been studied in this study. Screening brain cells stained with Rhodamine-123 dye showed that administration of Ca(OH)_2_NPs caused a marked damage to mitochondria as manifested by the high reduction in the emitted fluorescent light (Fig. 3). This result supports findings of Mohamed study which demonstrated that Ca(OH)_2_NPs are genotoxic and can cause mitochondrial damage and even neurodegenerative diseases like Alzheimer's. Brain tissue exposed to CaTiO_3_NPs showed less damage, but still some harm to mitochondria^10^. Meanwhile, administration of Y_2_O_3_NPs alone or with Ca(OH)_2_NPs and CaTiO_3_NPs showed no remarkable decrease in emitted fluorescent light and appeared safe for mitochondria confirming the protective effect of Y_2_O_3_NPs (Fig. 3).

## Conclusion

From above findings, it is concluded that administration of Ca(OH)_2_NPs alone induced the highest genomic DNA damage, ROS generation, disruption of mitochondrial membrane potential and inflammation, while administration of CaTiO_3_NPs alone had less toxic effects than Ca(OH)_2_NPs. Contrary, administration Y_2_O_3_NPs alone did not alter ROS generation, inflammatory genes expression and mitochondrial membrane potential. More interestingly, coadministration of Y_2_O_3_NPs with Ca(OH)_2_NPs and CaTiO_3_NPs alleviated the Ca(OH)_2_NPs and CaTiO_3_NPs induced genotoxicity, disruption of mitochondrial membrane potential and inflammation. However, single dose of nanoparticles, single tissue and limited techniques were used in this study, therefore further studies are necessary to fully understand the possibility of using Y_2_O_3_NPs to overcome Ca(OH)_2_NPs and CaTiO_3_NPs induced toxicity using different doses and more techniques in various organs.

### Supplementary Information

Supplementary Figure S1.

## Data Availability

The datasets used and/or analyzed during the current study are available from the corresponding author on reasonable request.
