# Supplementary figures and images for: Yttrium oxide nanoparticles ameliorates calcium hydroxide and calcium titanate nanoparticles induced genomic DNA and mitochondrial damage, ROS generation and inflammation
Source: Sci Rep. 2024 Jun 6;14:13015. doi: 10.1038/s41598-024-62877-4 (PMC11156978; doi:10.1038/s41598-024-62877-4)

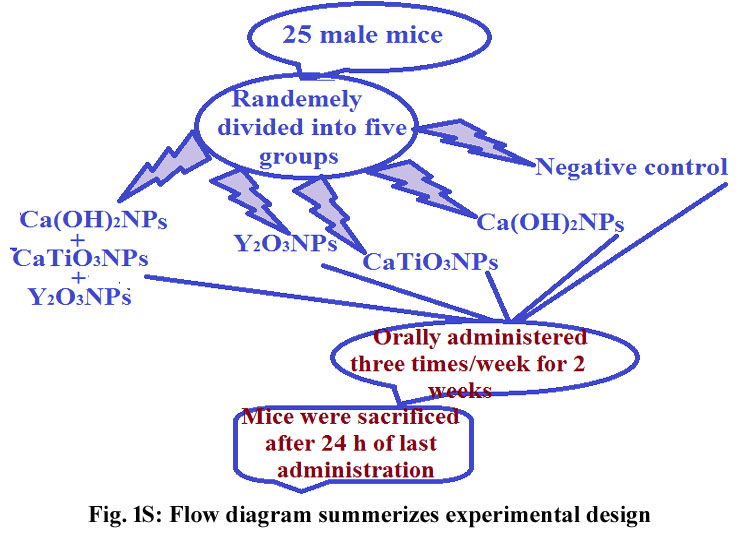

Supplement: Supplementary file 1 — Supplementary Figure S1. [file 41598_2024_62877_MOESM1_ESM.jpg]
